# Supplementary material for: An efficient and robust ABC approach to infer the rate and strength of adaptation
Source: G3 (Bethesda). 2024 Feb 14;14(4):jkae031. doi: 10.1093/g3journal/jkae031 (PMC11090462; doi:10.1093/g3journal/jkae031)
Supplement: jkae031_Supplementary_Data [file jkae031_supplementary_data.pdf]

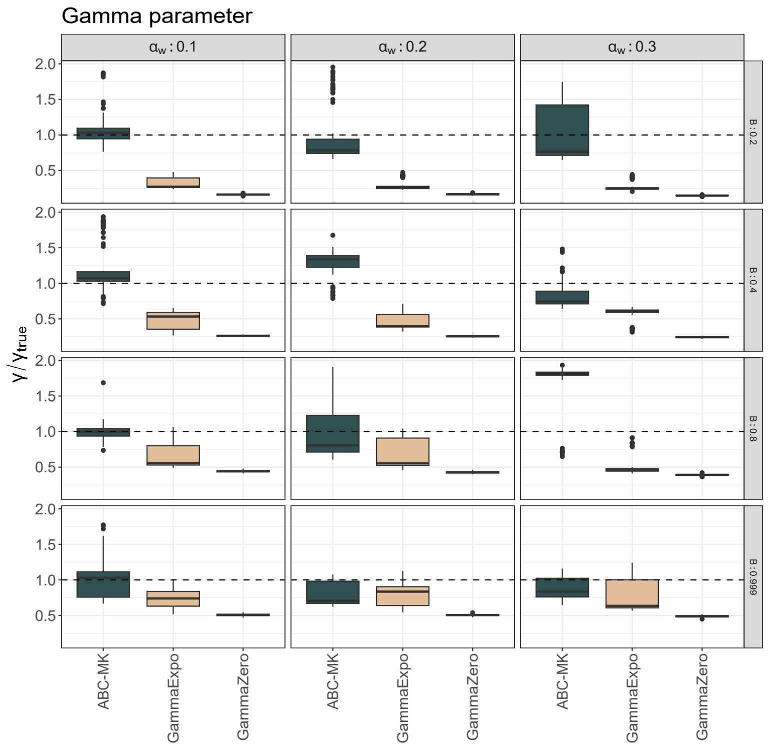

Figure 1 ABC-MK and Grapes DFE negative selection coefficient (γ) inference on the equilibrium simulations.

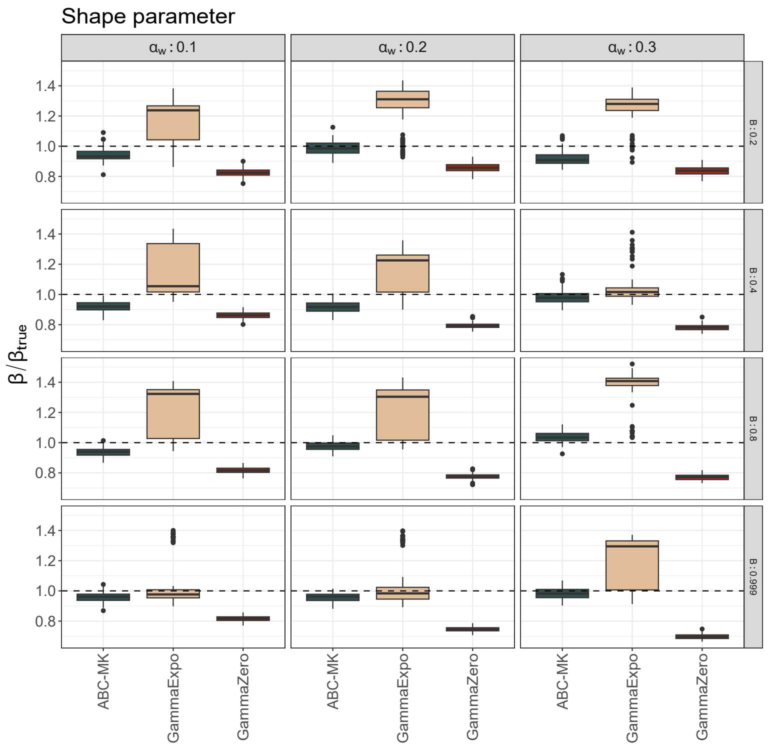

Figure 2 ABC-MK and Grapes DFE shape (β) inference on the equilibrium simulations.

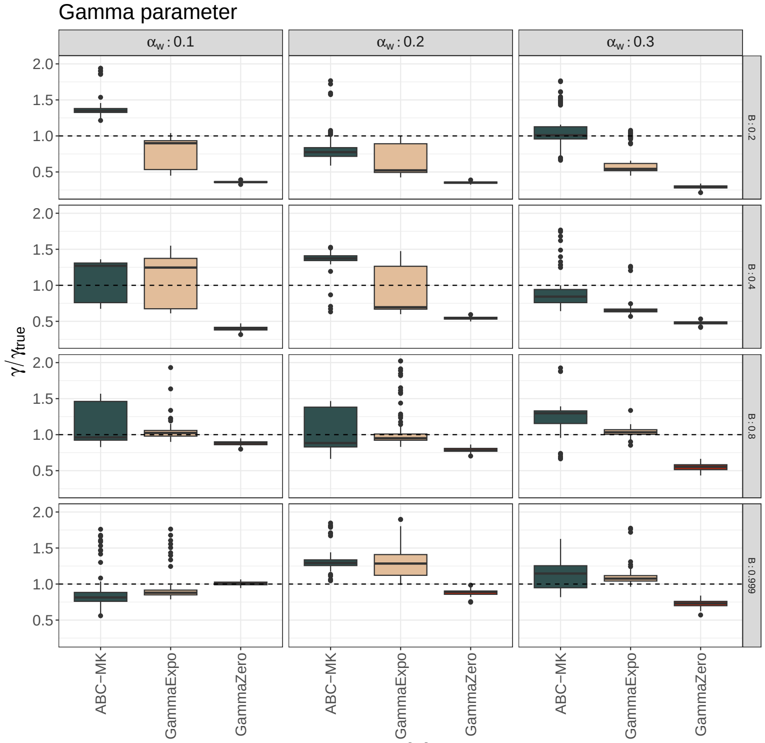

Figure 3 ABC-MK and Grapes DFE negative selection coefficient (γ) inference on Tennessen *et al.* (2012) .

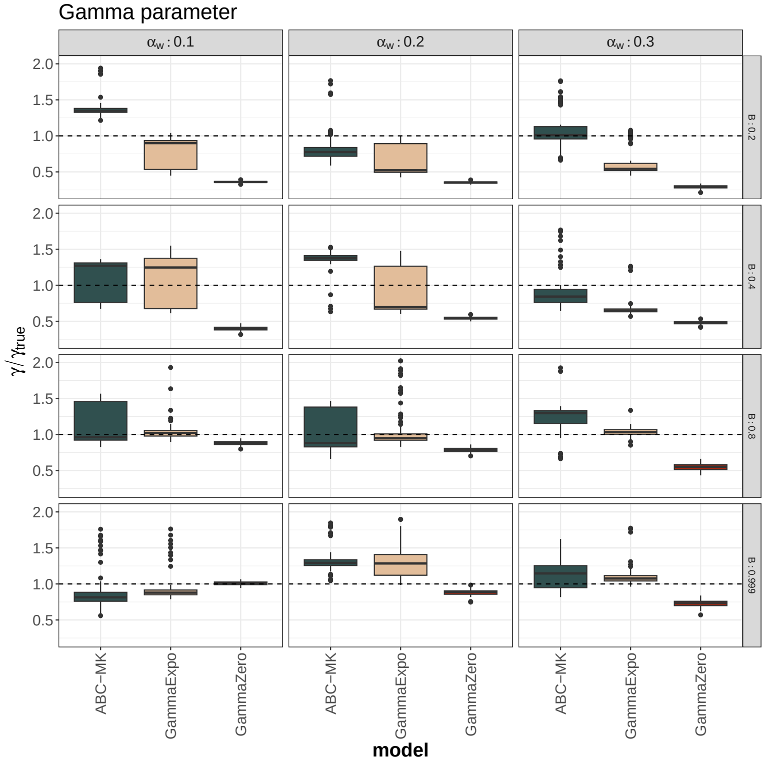

Figure 4 ABC-MK and Grapes DFE shape (β) inference on Tennessen *et al.* (2012) simulations.

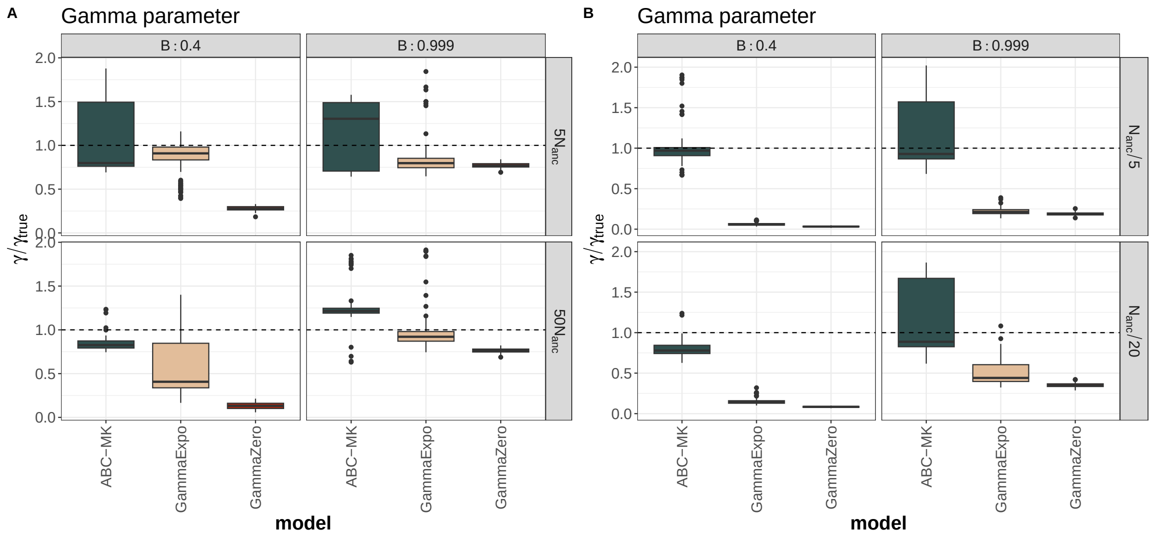

Figure 5 ABC-MK and Grapes comparison of the two-epoch model simulations negative selection coefficient (γ). A. Two-epoch expansion simulations. B. Two-epoch bottleneck simulations.

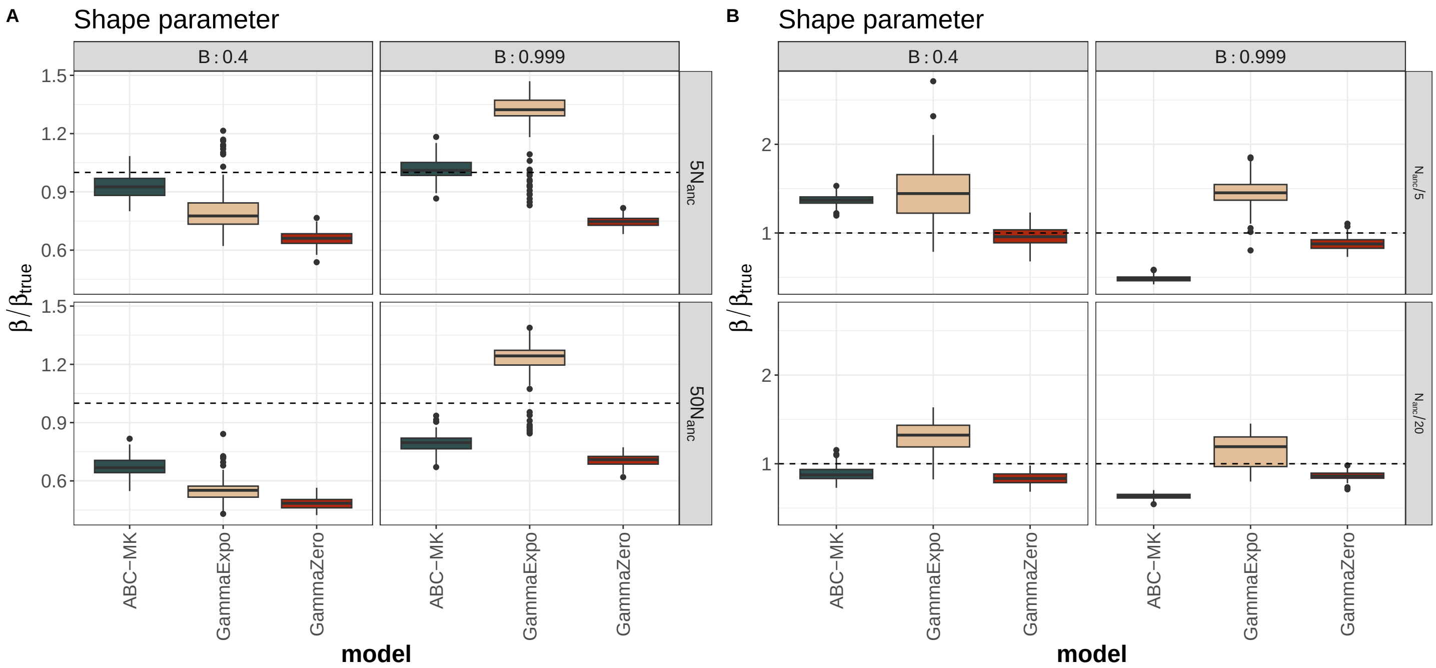

Figure 6 ABC-MK and Grapes comparison of the two-epoch model simulations. DFE shape (β) inference. A. Two-epoch expansion simulations. B. Two-epoch bottleneck simulations.

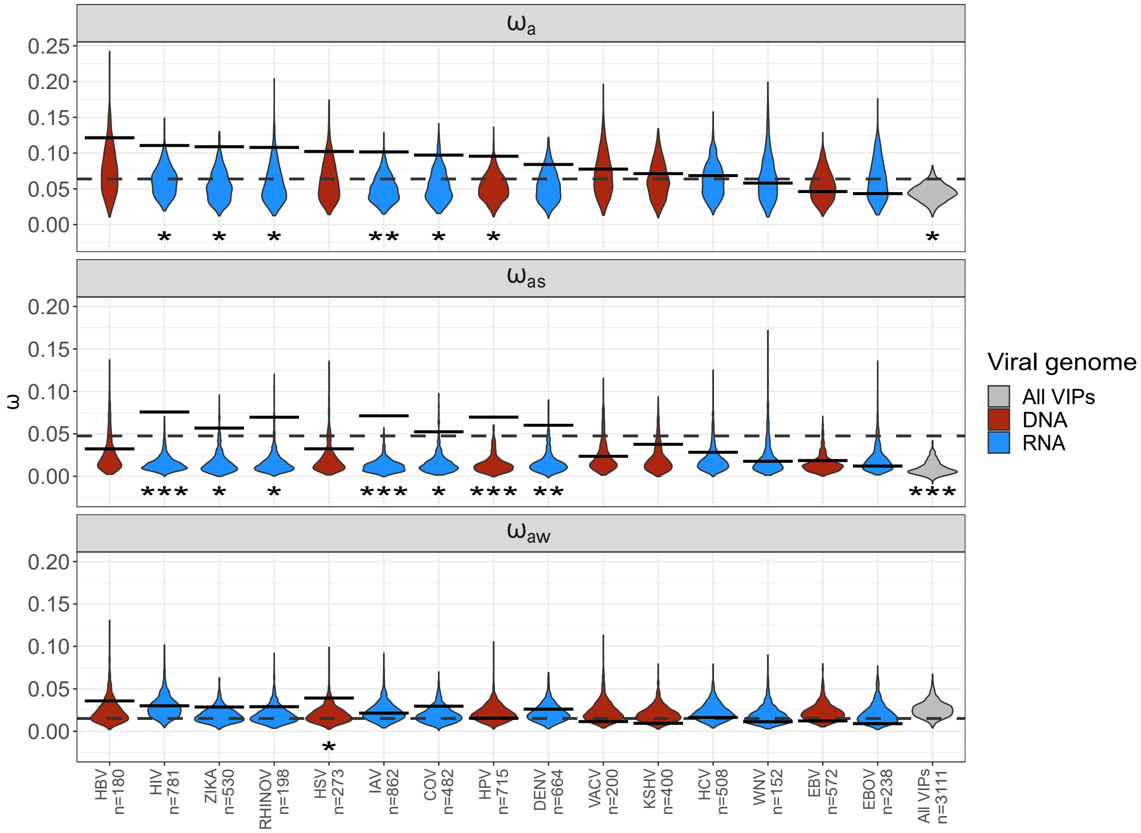

Figure 7 ABC-MK ω_a inference on different RNA and DNA-VIPs categories. Violin plots and solid lines represent inferences on non-VIP bootstrapped datasets and VIP categories according to the virus interaction. The dashed line is the overall VIPs inference.

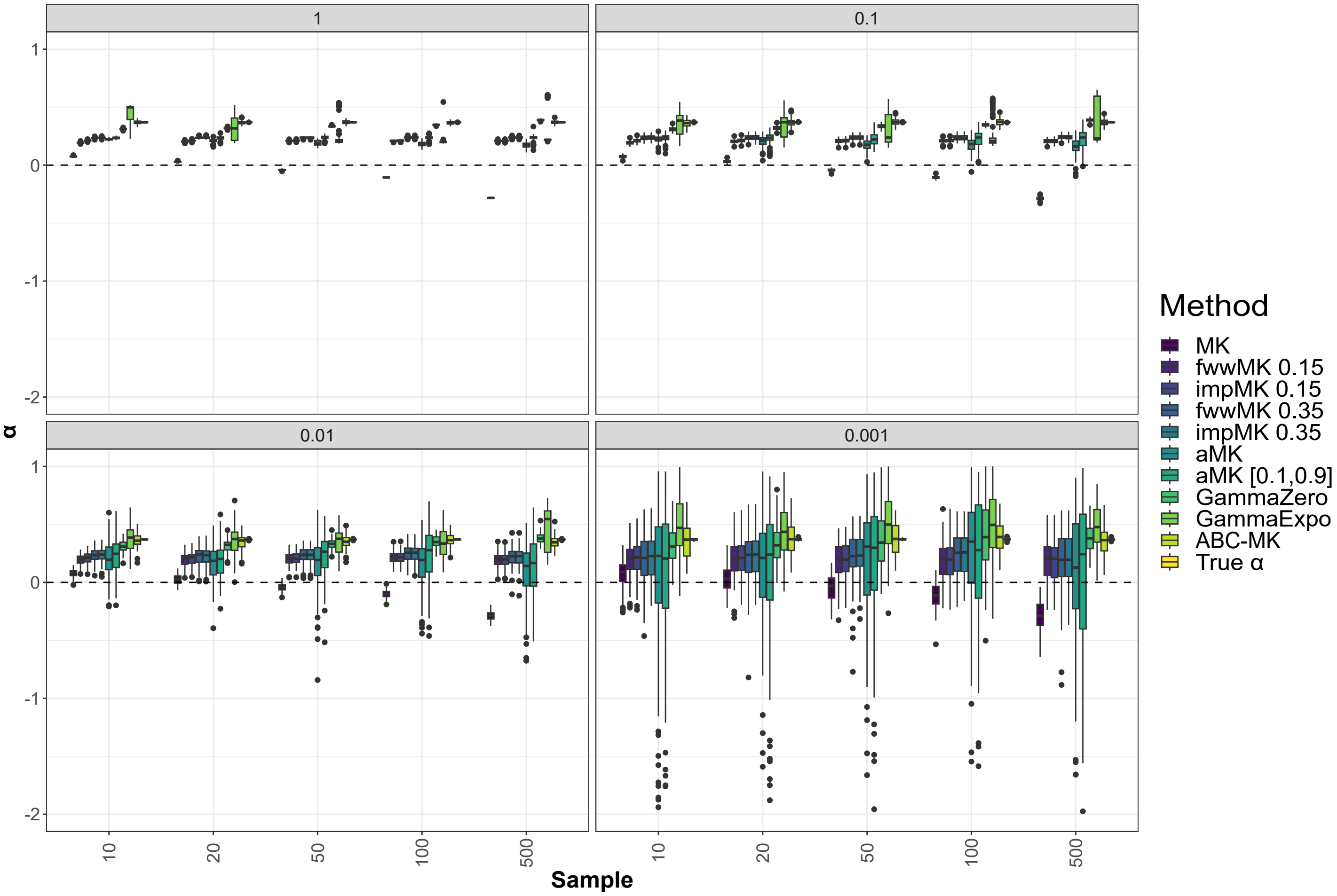

Figure 8 Effect of the sampling size and total number of polymorphic sites analyzed on several MK-tests. Each panel represents a proportion of the total number of simulations performed ABC-MK is robust to a low number of polymorphic sites and sampling size. Grapes GammaExpo became unstable depending on the sampling size and the total number of polymorphic sites.

Table 1 Mode and 95% CI of ω_{aW}, ω_{aS}, ω_a and p-values in human datasets.

Dataset	Viral genome	ω_{aW}	ω_{aS}	ω_a	P-value ω_{aW}	P-value ω_{aS}	P-value ω_a
RHINOV	RNA	0.029 [-0.027-0.173]	0.07 [-0.027-0.173]	0.108 [0.029-0.219]	0.049	0.018	0.161
HIV	RNA	0.03 [-0.01-0.159]	0.076 [-0.015-0.139]	0.111 [0.058-0.191]	0.014	0.001	0.396
HBV	DNA	0.036 [-0.018-0.2]	0.032 [-0.016-0.177]	0.121 [0.023-0.257]	0.092	0.265	0.176
ZIKA	RNA	0.029 [-0.01-0.168]	0.057 [-0.016-0.144]	0.109 [0.045-0.21]	0.015	0.024	0.130
IAV	RNA	0.021 [-0.015-0.129]	0.071 [-0.004-0.13]	0.102 [0.058-0.164]	0.005	0.001	0.496
HPV	DNA	0.015 [-0.013-0.122]	0.07 [-0.007-0.134]	0.096 [0.058-0.163]	0.017	0.001	0.731
COV	RNA	0.03 [-0.021-0.146]	0.052 [-0.009-0.146]	0.097 [0.029-0.193]	0.044	0.029	0.149
DENV	RNA	0.026 [-0.012-0.118]	0.06 [-0.009-0.126]	0.084 [0.045-0.161]	0.102	0.010	0.296
HSV	DNA	0.039 [-0.022-0.184]	0.032 [-0.021-0.146]	0.102 [0.006-0.222]	0.089	0.170	0.046
WNV	RNA	0.011 [-0.005-0.102]	0.018 [-0.006-0.104]	0.058 [0.006-0.145]	0.537	0.437	0.778
KSHV	DNA	0.009 [-0.011-0.115]	0.038 [-0.01-0.113]	0.071 [0.014-0.158]	0.341	0.147	0.909
HCV	RNA	0.016 [-0.006-0.122]	0.028 [-0.011-0.101]	0.068 [0.011-0.153]	0.382	0.187	0.726
VACV	DNA	0.011 [-0.01-0.109]	0.024 [-0.005-0.119]	0.078 [0.01-0.16]	0.367	0.356	0.882
EBV	DNA	0.012 [-0.007-0.107]	0.019 [-0.008-0.08]	0.046 [0.005-0.137]	0.619	0.251	0.864
EBOV	RNA	0.009 [-0.009-0.095]	0.012 [-0.007-0.086]	0.043 [-0.0-0.129]	0.706	0.661	0.888
All VIPs	DNA / RNA	0.015 [-0.014-0.088]	0.048 [-0.004-0.093]	0.064 [0.039-0.12]	0.050	0.001	0.900

Table 2 Sample size and total number of polymorphic sites

	Percentage of total simulated sites			
Sample size	1	0.1	0.01	0.001
10	179585	17962	1801	181
20	223487	22308	2240	224
50	286572	28654	2869	286
100	339035	33905	3388	336
500	482656	48270	4821	482

Literature cited

Tennessen JA, Bigham AW, O'Connor TD, Fu W, Kenny EE, Gravel S, McGee S, Do R, Liu X, Jun G *et al*. 2012. Evolution and Functional Impact of Rare Coding Variation from Deep Sequencing of Human Exomes. Science. 337:64–69.
